# Decoding the relationship of GFAP in serum with Dementia

**DOI:** 10.1002/alz70856_105434

**Published:** 2026-01-07

**Authors:** Alicia Uceda‐Heras, Iván Burgueño‐García, Paloma Ruiz‐Valderrey, Laura Saiz, María José López‐Martínez, Alberto Rabano

**Affiliations:** ^1^ Alzheimer's Center Reina Sofia‐CIEN Foundation, Madrid, Madrid, Spain; ^2^ Reina Sofia Foundation Alzheimer Centre, CIEN Foundation, ISCIII, Madrid, Madrid, Spain

## Abstract

**Background:**

Astrocytes are an essential glial cell which express GFAP, a marker of astrocytic activation and structural integrity. A recent study by our group links serum GFAP levels to tau pathology in Alzheimer's disease (AD), suggesting its value as a diagnostic biomarker.

Our aim is to examine the GFAP levels in serum (GFAPs) in correlation with various neuropathological variables, specifically focusing on the medial temporal lobe (MTL).

**Method:**

In 156 brains from the VARS dementia cohort, we analyzed a set of neuropathological variables including macroscopic data and classification and staging criteria for AD, Lewy Body Pathology (LBP), Cerebrovascular Disease (VD), Hippocampal sclerosis (HS), and TDP‐43 pathology (LATE).

**Result:**

First, we observed a correlation between GFAPs and tissue GFAP immunostaining in the entorhinal cortex (r=0.211, *p* <0.05). Then, we found a correlation of GFAPs with brain weight (r=‐0.366, *p* <0.001), NIA A (r=0.262, *p* <0.01), NIA B (r=0.416, *p* <0.001), NIA C (r=0.256, *p* <0.01), LPC classification (r=0.218; *p* <0.01), and with the presence of more combined pathologies with high burden (r=0.28, *p* <0.001). Besides, we observed a trend of increasing GFAPs values with higher stages of MTL atrophy, Braak tau, HS, and LATE. However, no significant correlation was detected between GFAPs and VD. Finally, a lineal regression model showed that NIA B (*p* <0.001), HS (head of the hippocampus) (*p* <0.05), and Braak α‐syn stages (*p* <0.05) were the best predictors for GFAPs.

**Conclusion:**

Our study shows that GFAP in serum correlates with neuropathological variables, specially with tissue tau pathology, supporting the potential of GFAP as a diagnostic biomarker for AD and related pathologies, except for cerebrovascular disease.

